# Cost-benefit analysis of VKA versus NOAC treatment in German patients with atrial fibrillation utilizing patient self-testing

**DOI:** 10.36469/9774

**Published:** 2019-08-07

**Authors:** Roland Diel, Niklas Lampenius

**Affiliations:** 1 Institute for Epidemiology, University Medical Hospital Schleswig-Holstein, Kiel, Germany and Institution for Statutory Accident Insurance and Prevention in the Health and Welfare Services (BGW), Hamburg, Germany; 2 Department of Accounting and Finance, University of Hohenheim, Stuttgart, Germany

**Keywords:** patient self-testing, noac, vka, anticoagulation, sensitivity analysis, cost-benefit analysis

## Abstract

**Background:** Clinical complications of long-term anticoagulation in patients with atrial fibrillation cause significant morbidity and have a substantial economic impact on the healthcare system.

**Objective:** To assess the cost-benefit by implementing patient self-testing (PST) in German patients anticoagulated with vitamin K antagonists (VKA) compared to treatment with the new oral anticoagulant drugs (NOAC) apixaban, dabigatran, edoxaban, and rivaroxaban.

**Methods:** A deterministic decision-analytic model was developed simulating the number of major bleedings, ischemic strokes, and hemorrhagic strokes and their associated costs by utilizing PST compared to those of treatment with NOAC. Data on the rates of these adverse events in both groups during the 1st year of treatment was taken from the NOAC approval studies. Direct costs were evaluated from the perspective of the Statutory Health Insurance (SHI) considering the use of resources directly related to PST testing and costs incurred by hospital treatment of the adverse events. Univariate sensitivity analysis was performed to examine the extent to which our calculations were affected by varying the parameters considered in our model within plausible extremes. To capture the interactions between multiple inputs, we also provided a probabilistic sensitivity analysis (PSA).

**Results:** When achieving an average time in therapeutic range (TTR) of 78%, implementing PST in VKA patients reduces cost per patient compared to NOAC treatment between €603.38 [USD 681.52] (edoxaban) and €762.64 [USD 861.40] (rivaroxaban) during the 1-year observation period. In line with the TTR increase, the initially higher number of adverse events per VKA patient compared to NOAC-treated patients in the approval studies becomes largely aligned; the difference in associated hospital costs per patient in the NOAC groups is then only €1.03 [USD 1.16] (in favor of dabigatran), €23.41 [USD 26.44] (in favor of apixaban), €0.53 [USD 0.60] (in favor of edoxaban) and €52.62 [USD 59.43] (in favor of VKA anticoagulation in the rivaroxaban group).

In PSA, implementation of self-management results on average in a cost saving between €619.20 [USD 699.39] and €785.24 [USD 886.93] per VKA patient in favor of the SHI. Under all reasonable assumptions, PST remains constantly less expensive irrespective of which NOAC is administered.

**Conclusion:** Implementing PST in German VKA patients may significantly reduce SHI expenditures compared to utilizing NOAC.

## Introduction

Atrial fibrillation (AF) increases the risk of stroke by a factor of 4–5 and accounts for almost 15% of all ischemic strokes.^1^ One in four middle-aged adults in Europe and the US is expected to develop AF, and by 2030, up to 7 million AF patients are anticipated in the European Union.^2^ Several studies have demonstrated that the risk of stroke is reduced by oral anticoagulant therapy with vitamin K antagonists (VKA), especially warfarin and phenprocoumon, which were the only oral anticoagulants available until a few years ago for primary and secondary prevention of thromboembolic events.

Currently, the non-vitamin K oral anticoagulants (NOAC) dabigatran, rivaroxaban, apixaban, and edoxaban are approved as potential alternatives of VKA treatment.^3–7^ As some meta-analyses suggest superiority of NOAC treatment versus VKA with respect to reduction of thromboembolisms and bleeding complications, the use of NOAC is recommended in the 2016 European Society of Cardiology (ESC) guidelines as first line therapy for anticoagulation in atrial fibrillation.^1^

However, one major point of criticism as stated by the Drug Commission of the German Medical Association^8^ is the unexpected short mean “time in therapeutic range” (TTR) of the International Normalized Ratio (INR), a standardized value to measure the required prolongation of prothrombin time, in the warfarin control groups of all NOAC approval studies.^3–7^ Thus, the higher rates of adverse vascular events in VKA patients investigated in those studies may partly be explained by the low mean TTR ranging between 55% (rivaroxaban) and 66% (edoxaban). In contrast, those VKA patients who utilize patient self-management (PST) during their VKA treatment usually have a significantly higher TTR of about 78%.^9^

In a cohort study of German AF patients using claim data of the most representative German SHI, the “Allgemeine Ortskrankenkassen” (AOK Health Insurance Fund) NOAC exposure was associated with significantly higher incidence rate ratios for death or non-specified strokes, myocardial infarction and severe bleeding suggesting that NOAC therapy doesn’t seem to be more effective and safer than a VKA therapy in “real life.”^10^ In a recently published Danish study^11^ on the treatment of AF patients, self-managed VKA treatment was associated with a significantly lower risk of all-cause and ischemic strokes compared to treatment with NOAC, whereas no significant differences were observed for major bleeding and mortality.

Indeed, there is evidence^12–14^ that an increase of TTR in patients under VKA therapy by patient self-testing (PST) is associated with a lower frequency of the most severe three adverse events, namely ischemic and hemorrhagic stroke and major bleedings. Based on a large database of 67,077 Veterans Health Administration patients anticoagulated with VKA, *Rose* *et al*.^15^ simulated the number of adverse events and their associated costs and utilities, both before and after various degrees of improvement in percent time in TTR, following a 2-year time horizon. There was demonstrated improvement in TTR by 10% with a prevented 2,087 events, gained 1,606 quality-adjusted life-years, and saved $29.7 million from the payer’s perspective.

Derived from *Rose*’s approach we defined mathematical formulas that allowed us to simulate the effects on the number of adverse events in the warfarin control groups of the original NOAC approval studies. We calculated the number of events that occurred when the original TTR in the studies would now uniformly increase to a mean TTR of 78%^9^; a figure which has been shown to reflect the reality in PST.

In a second step we assessed the costs of NOAC and VKA treatment of AF patients when PST^16^ was implemented for the first year under country specific conditions from the perspective of the German SHI.

Our aim was to examine the possible economic advantages of implementing PST in VKA patients compared to administering NOAC, when supervised by a properly trained general practitioner (GP). Since in Germany the short-acting warfarin is only rarely prescribed replaced it in our model of long-acting phenprocoumon, which is consumed by 98% of the German VKA patients^17^ and allows a higher stability of plasma concentrations.^18^

## Ethical considerations

Ethical approval was not necessary as only publicly available secondary data were used.

## Material and Methods

### a) Model approach

Our model was parametrized by data on the rates per patient and year of three main adverse events in the study populations of the four NOAC approval publications.

We assume that a relevant increase in the TTR is possible by PST under the supervision of a GP. Only costs to be carried by the SHI that arise on the legal basis of the German social security statute book (SGB) V are included. These are the costs of medication (NOAC and phenprocoumon), aids (CoaguChek^®^ system, test strips and lancets, see^16^ in accordance with §31 SGB V), costs of training on the proper use of the test system, the costs for outpatient primary care and laboratory costs and the result of hospital stays as a result of adverse events. The frequency of the three adverse events in both comparison groups (patients with NOAC or VKA) reported in the NOAC approval studies^3–7^ is standardized to one year due to the different duration of the approval studies in order to allow for comparability. Due to the lack of empirical data, the model assumes that the adverse events occur at the end of the first year of comparison and, due to their severity, require hospitalization.

The outpatient costs refer to the National Association of Statutory Health Insurance Physicians’ Uniform Assessment Standard (EBM),^19^ as of January 2019 and – with respect to the reimbursement of medical devices and other aids for performing patient self-management – to the agreement with the National Association of Statutory Health Insurance Funds (“Spitzenverbände der Krankenkassen”) of February 2002. Costs arising from hospitalization and from prescribing anticoagulants are based on the G-DRG (German diagnosis related groups) system in the most recent version (2019) and the Pharmacy Sales Prices (Red List [Rote Liste^®^] 2019), respectively. Any discounts granted by the manufacturers of NOAC to some insurances are not taken into account for reasons of transparency.

### b) Model structure

**Figure 1.**
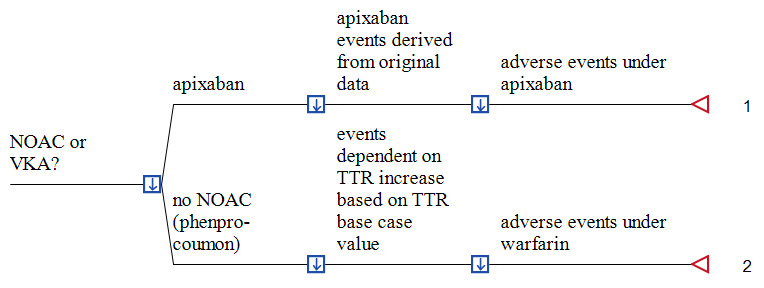
Section of the probabilistic cost-benefit decision tree of administering apixaban versus VKA plus PST in AF patients Formula 1: dist_cApix + dist_cPausch + dist_cIStroke*dist_noIS_Apix + dist_cHStroke*dist_noHS_Apix + dist_cMajorBL*dist_noMajorBL_Apix Formula 2: dist_cPausch + (dist_cCoasyst/dist_pDepr) + dist_cCoatrain + dist_cCoalab + (dist_cCoalanc*57) + (dist_cCoastrip*57) + dist_cMarcu + dist_noIS_War_Apix*dist_cIStroke - dist_noIS_War_Apix*(0.0134 + 0.6754*DELTA_Apix)*dist_cIStroke + dist_noHS_War_Apix*dist_cHStroke - dist_noHS_War_Apix*(0.0101 + 1.4282*DELTA_Apix)*dist_cHStroke + dist_noMajorBL_War_Apix*dist_cMajorBL - dist_noMajorBL_War_Apix*(0.0101 + 1.4282*DELTA_Apix)*dist_cMajorBL Legend: A decision node (square) indicates a choice facing the decision maker or the consequences of a decision. Branches from a chance node (circles) represent the possible outcomes of an event; terminal nodes (triangles) denote the endpoints of a scenario and are assigned the cost of a prior series of actions and events. The arrows in the decision notes pointing downwards demonstrate that the optimal path of the model is that with the lowest total cost. The prefix “dist” means distribution of the cost parameters or number of events as described in Table 1.

A deterministic, patient-based decision-analytic model was developed, simulating the incremental costs of using one of the NOAC, compared to conventional VKA treatment, with and without PST. The perspective taken is that of the SHI itself (see Figure 1 for an example using apixaban). In this figure, the decision tree simulates the direct costs of the two alternative anticoagulation strategies per patient until the end of the first year for one year. We used TreeAge Software (TreeAge Inc. Williamstown MA, USA) for model building and analysis and examined our inputs over a wide range in sensitivity analyses to identify influential factors that would alter the base-case findings.

Univariate sensitivity analysis was performed using all variables to examine the extent to which our calculations are affected by varying selected assumptions. Variation was done using either a) the lower and upper bounds of the parameter if present or b) the lower and upper bounds of a parameter’s 95% confidence interval. Where this is not applicable, we vary parameter values by ± 20% of the base-case value according to international practice, unless stated otherwise.

Furthermore, in order to capture the interactions between multiple inputs we provide a probabilistic sensitivity analyses (PSA) by assigning an appropriate statistical (probability) distribution to all input parameters, from which values are randomly drawn in a 2nd order Monte-Carlo simulation (n=1,000). All costs are reported in 2019 Euros (€). Values were calculated in EUR and the average exchange (1 EUR = 1.295 USD for 01.01.19 to 17.07.2019, retrieved from European Central Bank (ECB) at https://www.ecb.europa.eu/stats/policy_and_exchange_rates/euro_reference_exchange_rates/html/eurofxref-graph-usd.en.html) was used to convert the EUR amounts to USD.

Input parameters are shown together with their probabilistic distributions in Table 1.

**Table 1:** Input for cost-benefit analysis

**Variables category**	**Variable name**	Distribution*	**Value (base case)**	**Relative change (range)**	**Reference**
Drug costs of apixaban per year	cRiva	triangular	€1253.74	(± 20%) €1002.99-€1504.49	Red List [Rote Liste^®^] 2019
Drug costs of dabigatran per year	cDabi	triangular	€1197.77	(± 20%) (€958.21 - €1437.32)	Red List [Rote Liste^®^] 2019
Drug costs of edoxaban per year	cEdox	triangular	€1099.48	(± 20%) €870.79-€1306.18	Red List [Rote Liste^®^] 2019
Drug costs of rivaroxaban per year	cRiva	triangular	€1194.59	(± 20%) €955.67- €1433.51	Red List [Rote Liste^®^] 2019
Costs of phenprocoumon per year	cMarcu	triangular	€66.78	(± 20%) €53.42-€80.14	Red List [Rote Liste^®^] 2019
Costs of hemorrhagic stroke per event	cHStroke	uniform	€5480.52	(± 20%) €4384.42-€6576.62	calculated
Costs of ischemic stroke per event	cIStroke	uniform	€4948.78	(± 20%) €3595.02-€5938.54	calculated
Costs of major bleeding per event	cMajorBL	uniform	€3814.39	(± 20%) €3051.51-€4577.27	calculated
Costs of care by settled physician per year	cPausch	uniform	€113.40	(± 20%) €90.72-€136.08	EBM [19]
Costs of laboratory controls by physician for self-managing patients per year	cCoalab	triangular	€3.6	(± 20%) €2.88-€4.32	EBM [19]
Costs of training when using Coaguchek	cCoatrain	uniform	€166.64	(± 20%) €84-€150	Own interviews
Costs of one CoaguChek test strip	cCoastrip	uniform	€2.86	(± 20%) €2.288-€3.43	Internet best price
Costs of CoaguChek INR device	cCoasyst	uniform	€671.35	(± 20%) €537.08-€805.62	Internet best price
Period of depreciation	pDepr	triangular	5 yr	3-10 yr	German depreciation guidelines
TTR difference between approval study and 78%	DELTA_Apix	-	0.16	-	calculated
TTR difference between approval study and 78%	DELTA_Dabi	-	0.14	-	calculated
TTR difference between approval study and 78%	DELTA_Edox	-	0.13	-	calculated
TTR difference between approval study and 78%	DELTA_Riva	-	0.23	-	calculated
No. of hemorrhagic strokes per patient at risk and year under apixaban	noHS_Apix	uniform	0.0024	(95% CI) 0.0014-0.0034	[3]
No. of hemorrhagic strokes per patient at risk and year under dabigatran	noHS_Dabi	uniform	0.0010	(95% CI) 0.0002-0.0018	[4]
No. of hemorrhagic strokes per patient at risk and year under edoxaban	noHS_Edox	uniform	0.0026	(95% CI) 0.0014-0.0038	[7]
No. of hemorrhagic strokes per patient at risk and year under rivaroxaban	noHS_Riva	uniform	0.0026	(95% CI) 0.0013-0.0039	[5,6]
No. of hemorrhagic strokes per warfarin patient at risk and year in the apibaxan trial	noHS_War_Apix	uniform	0.0047	(95% CI) 0.0033-0.0061	[3]
No. of hemorrhagic strokes per warfarin patient at risk and year in the dabigatran trial	noHS_War_Dabi	uniform	0.0038	(95% CI) 0.0022-0.0054	[4]
No. of hemorrhagic strokes per warfarin patient at risk and year in the edoxaban trial	noHS_War_Edox	uniform	0.0047	(95% CI) 0.0031-0.0063	{7]
No. of hemorrhagic strokes per warfarin patient at risk and year in the rivaroxaban trial	noHS_War_Riva	uniform	0.0042	(95% CI) 0.0025-0.0059	[5,6]
No. of ischemic strokes per patient at risk and year under apixaban	noIS_Apix	uniform	0.0097	(95% CI) 0.0077-0.0177	[3]
No. of ischemic strokes per patient at risk and year under dabigatran	noIS_Dabi	uniform	0.0092	(95% CI) 0.0068-0.0116	[4]
No. of ischemic strokes per patient at risk and year under edoxaban	noIS_Edox	uniform	0.0125	(95% CI) 0.0092-0.0148	[7]
No. of ischemic strokes per patient at risk and year under rivaroxaban	noIS_Riva	uniform	0.012	(95% CI) 0.0092-0.0148	[5,6]
No. of ischemic strokes per warfarin patients at risk and year in the apixaban trial	noIS_War_Apix	uniform	0.0105	(95% CI) 0.0148-0.0126	[3]
No. of ischemic strokes per warfarin patient at risk and year in the dabigatran trial	noIS_War_Dabi	uniform	0.012	(95% CI) 0.0092-0.0148	[4]
No. of ischemic strokes per wafarin patient at risk and year under edoxaban	noIS_War_Edox	uniform	0.0125	(95% CI) 0.0099-0.0151	[7]
No. of ischemic strokes per warfarin patient at risk and year in the rivaroxaban trial	noIS_War_Riva	uniform	0.0134	(95% CI) 0.0104-0.0164	[5,6]
No. of major bleedings per patient at risk and year under apixaban	noMajorBL_Apix	uniform	0.0407	(95% CI) 0.0366-0.0488	[3]
No. of major bleedings per patient at risk and year under dabigatran	noMajorBL_Dabi	uniform	0.0311	(95% CI) 0.0267-0.0355	[4]
No. of major bleedings per patient at risk and year under edoxaban	noMajorBL_Edox	uniform	0.0275	(95% CI) 0.0237-0.0313	[7]
No. of major bleedings per patient at risk and year under rivaroxaban	noMajorBL_Riva	uniform	0.0339	(95% CI) 0.0292-0.0386	[5,6]
No. of cases of major bleedings per warfarin patient at risk and year in the apibaxan trial	noMajorBL_War_Apix	uniform	0.0601	(95% CI) 0.0552-0.065	[3]
Number of major bleedings per warfarin patient at risk and year in the dabigatran trial	noMajorBL_War_Dabi	uniform	0.0336	(95% CI) 0.029-0.0382	[4]
Number of cases of major bleedings per warfarin patient at risk and year in the edoxaban trial	noMajorBL_War_Edox	uniform	0.0343	(95% CI) 0.03-0.0313	[7]
No. of cases of major bleedings per warfarin patient at risk and year in the rivaroxaban trial	noMajorBL_War_Riva	uniform	0.0317	(95% CI) 0.271-0.0363	[5,6]

*in probabilistic sensitivity analysis

### c) Model input

#### 1. Mathematical model

The information from Table 5 of *Rose*’s publication^15^ was utilized to calibrate a model that captures the influence of a change (delta, ∆) in TTR (∆TTR) on the number of ischemic strokes (IS) and major hemorrhages (MH) in relationship to those TTR-changes. As MH and hemorrhagic strokes (HS) were not assessed separately in *Rose*’s publication we assumed that the effect of TTR increase on MH and HS would be the same.

We standardized the data from *Rose et al*.^15^ to derive a linear dependency structure in a regression framework. Subsequently, we regressed TTR on IS and TTR on MS to derive a dependency structure using robust standard errors, where regression results are reported in Table 2.

**Table 2:** Regression results derived from the data of <italic>Rose</italic> et al.<sup>15</sup>

**Dependent Variable:**	**Ischemic Stroke**	**Major Bleedings**
Change in TTR (∆TTR)	0.6754*** (18.33)	1.4282*** (39.29)
Constant	0.0134** (3.23)	0.0101* (2.67)
N	6	6

***, **, * indicates significant estimates at the 0.01, 0.05, and 0.1 significance level with T-statistics in parentheses

The estimated coefficients were used to generalize the findings of *Rose et al*., i.e., we utilize the following relationship to estimate mean relative savings:

Relative Savings in IS = 0.0134 + 0.6754 * ∆TTR and

Relative Savings in MH = 0.0101 + 1.4282 * ∆TTR

Multiplying the relative savings in IS or relative savings in MH, resulting from a particular ∆TTR, with the baseline IS or MH defines the savings in IS and MH related to a particular ∆TTR.

To incorporate and compare results from different studies we standardize effects to a patient at risk in the respective study population using the following relationship:

Saving IS: Baseline of study (number per study patient at risk in % per year) * (0.0134 + 0.6754 * ∆TTR)

Saving MH: Baseline of study (number per study patient at risk in % per year) * (0.0101 + 1.4282 * ∆TTR).

#### 2. Epidemiological parameters (taken form the approval studies 3-7):

The risk of having an ischemic stroke (IS), hemorrhagic stroke (HS) or major bleedings (MB) in the NOAC approval studies are separately shown for the respective NOAC and the warfarin control group in Table 3, together with the mean time in therapeutic range (TTR) of the warfarin group members.

**Table 3.** Risk of having severe adverse events in the respective NOAC groups and their warfarin controls patients

NOAC	Risk of IS per year in %	Risk of HS per year	Risk of MB per year	VKA used in the respective NOAC study	Risk of IS per year	Risk of HS per year	Risk of MB per year	Mean TTR in%
Apixaban^3^	0.97	0.24	4.07	Warfarin	1.05	0.47	6.01	62.2 (no SD provided)
Dabigatran^4^	0.92	0.10	3.11	Warfarin	1.20	0.38	3.36	64.4 (no SD provided)
Edoxaban^5^	1.25	0.26	2.75	Warfarin	1.25	0.47	3.43	64.9 ± SD18.7
Rivaroxaban^6,7^	1.20	0.26	3.39	Warfarin	1.34	0.42	3.17	55.0 (no SD provided)

TTR: Time in therapeutic range; SD: Standard deviation; IS: Ischemic stroke: HS: Hemorrhagic stroke; MB: Major bleeding

#### 3. Economic parameters

3.1 Reimbursement in favor of hospitals (DRG case flat rates) of the three adverse events (ischemic insult, hemorrhagic insult and major bleeding) was calculated using the webgrouper of the University of Münster.^20^ We also considered the most common comorbidities in German AF patients as assessed in the study of Ujeyl et al.^21^ The revenue for treating an ischemic or hemorrhagic stroke and major bleedings was €4,948.78 [USD 5,589.65], €5,480.52 [USD 6,190.25] and € 3,814.39 [USD 4,308.35) respectively. Full details are provided in the Appendix.

3.2. Drug costs

3.2.1 Costs of the vitamin K antagonist phenprocoumon

The target range for treatment with VKA is in the INR range 2 to 3. In order to reach the target area, a saturation phase is required. Marcumar^®^ and the generics available in Germany contain 3 mg of phenprocoumon per tablet (Tbl.). The defined daily dose (DDD) of phenprocoumon is 3mg (one Tbl.) According to the German Rote Liste^®^ 2019 the price of 100 Tbl. is €17.89 [USD 20.21]; therefore, the costs per day amount to €0.1798 [USD 0.2031].

The scheme for phenprocoumon saturation in a normal weight adult with no hepatic dysfunction is as follows:

1st day: Three Tbl.: €0.54 [USD 0.61]

2nd day: Two Tbl.: €0.36 [USD 0.41]

3rd day: Two Tbl.: €0.36 [USD 0.41]

4th day: One Tbl.: €0.18 [USD 0.20]

5th day: Measuring the INR value and dosage according to the result.

If the INR value is above the therapeutic range on day five, there is an overdose and a medication break is taken for at least one day. Thereafter, treatment should be continued with half a tablet. After four days, the INR is measured again and the dose adjusted if necessary.

This means as a minimum in the first 9 days €1.8 [USD 2.03] (€1.44 [USD 1.63] plus €0.09 € [USD 0.10] x 4).

If the INR value is below the therapeutic range on day five, the saturation phase is extended by one day by adding two tablets. This is followed by the administration of 1.5 tablets for three days. After four days, the INR is measured again and the dose adjusted if necessary. This means a maximum of €2.61[USD 2.95] in the first 8 days (€1.44 [USD 1.63] plus €0.36 [USD 0.41] plus €0.27 [USD 0.30] x 3).

Since the usual maintenance dose for phenprocoumon is between 1.5 mg (half Tbl.) and 4.5 mg (1.5 Tbl.), an average value of 1 Tbl. is assumed.

3.2.2 Costs of NOAC (German “Red List” 2019)

3.2.2.1 Apixaban

The recommended daily dose of apixaban (Eliquis^®^ ) is 2 x 5 mg. 200 Tbl. of 5 mg (or even in the lower dose 2.5 mg) amount to (reimbursement amount by the GKV) €343.49 [USD 387.97]. The daily cost are €3.43 [USD 3.87]. The annual costs are €3.4349 [USD 3.88] x 365= €1,253.74 [USD 1,416.10].

3.2.2.2 Rivaroxaban

The recommended daily dose of Xarelto^®^ is 20mg. 98 Tbl. of 20 mg each (or 15 mg) cost €320.74 [USD 362.28]. The daily dose thus amounts to €3.27 [USD 3.69] (rounded). The annual costs are 365 x €3.27 [USD 3.69] = €1,194.59 [USD 1,349.29].

3.2.2.3 Edoxaban

The recommended daily dose of Lixiana^®^ is 60 mg once a day. 98 Tbl. of each 60 mg (or 30 mg) cost €292.25 [USD 330.10]. The daily dose costs €2.98 [USD 3.37] (rounded). The annual costs are 365 x €2.55 [USD 2.88] = €1,099.48 [USD 1,241.86].

3.2.2.4 Dabigatran

Dabigatran (Pradaxa^®^) 150 mg twice a day is the recommended daily dose. 180 hard capsules of 150 mg (or 110 mg) cost €295.45 [USD 333.71]; the daily dose (€295.34 [USD 333.59]: 90) thus costs €3.2815 [USD 3.7065]. The annual costs are 365 x €3.28 [USD 3.70] = €1,197.77 [USD 1,352.88]. Of note, also the reduced doses, e.g. for patients suffering from impaired kidney function, are not less expensive.

3.3 Cost of coagulation self-management

The measurement intervals for coagulation self-management are much closer than for measurements exclusively in primary care practice (see below). An autonomous self-adaptation of the phenprocoumon dose requires training, a sufficient stock of test strips and the presence of a (maintenance-free) test device.

According to §31 (3) SGB V, there is no copayment obligation of the insured persons for blood test strips. Since lancets, test strips and the measuring device according to § 31 (1) SGB V constitute an accepted aid, there is a claim for full reimbursement by the SHI. The costs of the necessary training are on average €106.64 [USD 120.45], varying between €84 [USD 94.88] and €150 [USD 169.43] in the German federal states (own investigation). Training costs for physicians and/or the entire practice team are not considered, since these are not carried by the SHI.

For the INR tests until the end of the first month (initial phase) 9 strips (in the first week 3 pieces, then 2 pieces per week) are needed; then 1 strip per week. A test strip costs €2.86 [USD 3.23] (2 x 24 test strips each €137.31 [USD 155.09] - as of 18.3.2019), of which a total of 57 (for 9 + 48 days) are required per year and thus a total of €163.06 [USD 184.18] (rounded). The annual costs of CoaguChek^®^ Softclix lancets is currently €9 [USD 10.17] for 50 pieces; therefore, the costs for 57 tests amount to €10.26 [USD 11.59] (57 x €0.18 [USD 0.20]).

Current best prices for the CoaguChek^®^ INRange System (PZN11296110) are €671.35 [USD 758.29] (current date 1.6.2019). The CoaguChek^®^-INR device is depreciated over 60 months, as is customary for medical measuring instruments (36 months to 10 years in sensitivity analysis).

3.4. Laboratory costs in the GP’s practice

According to the statements in the Federal Gazette, a total of 6 additional medical check-ups are accepted in medical practice (1st quarter in each case monthly, i.e., three times, and one time each in the 2nd up to the 4th quarter. As it is also necessary to check to what extent the INR value obtained with the CoaguChek^®^ INR system is in accordance with the laboratory value as a reference, it is generally assumed that the blood will be drawn at the GP’s practice and subsequently transferred to the collaborative laboratory. The laboratory community will receive €0.6 [USD 0.68] per examination in accordance with fee schedule position (GOP) 32113. Thus, costs of €3.60 [USD 4.07] (€0.6 [USD 0.68] [GOP 32113] x 6) incur at the expense of the SHI.

3.5. Costs of control by the GP

According to *Schnabel* et al.^22^ the median age of patients with AF in Germany is 52.2 ± 11 years. Thus, a GP can bill an amount of €13.20 [USD 14.91] per quarter for the age group 19-54 (GOP 03000). In addition, there is the remuneration of €14.07 [USD 15.89] per quarter (GOP 03220 as surcharge to GOP 03000) and the amount of €1.08 [USD 1.22] and for GOP 03222 (surcharge to GOP 03220). All in all, the GP will be paid flat rates of €113.40 [USD 128.09] (4 x €28.35 [USD 32.02]) per year.

3.6. Cost of NOAC control

According to the proposals of the 2nd edition of the Practical Guide of the European Heart Rhythm Association^23^ follow-up intervals are required for NOAC: first check one month after the initial prescription; in addition, then clinical controls ± every 3 months, a maximum of 6 months depending on patient factors such as age, renal function and comorbidities.

Nevertheless, in view of the frequent comorbidities in patients with AF, e.g. diabetes mellitus or arterial hypertensions, it would be unrealistic to assume that during the study period of one year NOAC patients would not see their doctor for whole quarters and that type and frequency of routine parameters to be investigated (blood count, liver and kidney values) differ from those of phenprocoumon patients. Therefore, the same costs, as for VKA patients, of €113.40 [USD 128.09] are assumed as payment for the GP per year. The costs for determining those routine parameters are not calculated separately for reasons of insignificance (determining the creatinine value, for example, amounts to only €0.25 [USD 0.28] and performing a blood count to only €0.5 [USD 0.56]) and are not considered in our model.

## Results

Achieving a mean TTR of 78% by implementing patient’s self- management (PST) is on average between €603.38 [USD 681.52] and €762.63 [USD 861.39] less costly in the first year per VKA patient, compared to utilizing NOAC (see Table 4 a-b). Assuming that an increase of the original mean TTR as reported in the respective approval studies by approximately 16 percentage points for apixaban, 14 points for Dabigatran, 13 points for edoxaban, and 23 points for rivaroxaban to the target of 78% can be achieved, the number of severe adverse events in VKA patients decreases, and accordingly the associated hospital costs become largely aligned. Thus, after TTR adjustment, the cost difference for those severe events per VKA and NOAC patient in the various groups is then only €1.03 [USD 1.16] (in favor of Dabigatran), €23.41 [USD 26.44] (in favor of apixaban), €0.53 [USD 0.60] (in favor of edoxaban) and €52.62 [USD 59.43] (in favor of VKA anticoagulation in the rivaroxaban group).

**Table 4a:** Costs of adverse effects: Adverse events costs according to NOAC approval studies (without TTR increase)

NOAC	Cost per patient (NOAC) in €	Cost per patient (VKA) in €	Difference to VKA per patient in €	Adverse events costs per patient (NOAC) in €	Adverse event costs per patient (VKA) in €	Difference of adverse events costs to VKA per patient in €
Rivaroxaban	1510.93	748.29	- 762.64	202.94	210.25	+ 7.31
Dabigatran	1480.81	768.64	- 712.17	169.64	208.37	+ 38.73
Apixaban	1583.54	837.78	- 745.76	216.40	306.97	+ 90.56
Edoxaban	1382.88	779.51	- 603.38	181.0	218.45	+ 37.45

**Table 4b:** Costs of adverse effects: Results after TTR increase to 78%

NOAC	Cost per patient (NOAC) in €	Cost per patient (VKA) in €	Difference to VKA per patient in €	Adverse events costs per patient (NOAC) in €	Adverse event costs per patient (VKA) in €	Difference of adverse events costs to VKA per patient in €
Rivaroxaban	1510.93	748.29	- 762.64	202.94	150.32	- 52.62
Dabigatran	1480.81	768.64	- 712.17	169.64	170.67	+ 1.03
Apixaban	1583.54	837.78	- 745.76	216.40	239.81	+ 23.41
Edoxaban	1382.88	779.51	- 603.38	181.0	181.54	+ 0.53

Univariate sensitivity analysis, in which all variables in the decision trees receive assigned values within their respective ranges, reveals that the drug costs of the single NOAC per patient and year have the highest impact on the absolute amount of cost savings resulting in a linear increase or decrease of 20% - the chosen lower upper and lower bounds of sensitivity analysis (Table 5 a-d). The break-even point when the costs of the NOAC strategy would fall below those of VKA treatment would only be achieved when the annual drug costs of the NOAC would be reduced between 55.43% (edoxaban; €485.1 [USD 547.92]/€1,088.46 [USD 1,229.42]) and 63.84% (rivaro-xaban; €431.96 [USD 487.90]/€1,194.59 [USD 1,349.29]).

Three other parameters still have moderate impact on the cost difference between the NOAC and VKA strategy: Varying the depreciation period for the expensive INR measurement device (CoaguChek^®^) between only 3 years and 10 years results in a difference of €156.65 [USD 176.94] in favor of the shorter period. Potential cost savings also depend on the amount of training costs that is mandatory for independently performing INR measurement and varies between €84 [USD 94.88] and €150 [USD 169.43]in the German federal states. Also, the relative small variation of the costs of one test strip by €0.57 [USD 0.64] results in additional or lower costs per VKA patient and year of €66.00 [USD 74.55]. In contrast, varying the G-DRG costs of hospitalization for treating the ischemic or hemorrhagic stroke or major bleeding as potential adverse events by ± 20% resulted in minor changes of total cost. This is because costs incurred for those adverse events would always change in parallel in both arms of the decision tree.

In PSA, i.e. under all reasonable assumptions, the costs of implementing PST for anticoagulation at the expense of the SHI saved on average was between €619.2 [USD 699.39] (edoxaban) and €785.24 [USD 886.93] (rivaroxaban, see Table 5). Here treatment with phenprocoumon remains constantly less expensive than treatment with one of the four NOAC.

**Table 5a:** Tornado diagram*: VKA under PST versus apixaban

**Variable Name**	**Variable Description**	**Variable lowest bound**	**Variable highest bound**	**Lowest cost value**	**Highest costs value**	**Spread^Ƭ^**	**Risk%^¥^**	**Cum Risk%**
cApix	Drug costs of apixaban per year in €	1002.99	1504.49	495.01	996.51	501.50	0.854	0.85
pDepr	Period of depreciation	3.00	10.00	656.25	812.90	156.65	0.083	0.94
cCoatrain	Costs of training when using Coaguchek in €	84.00	150.00	702.40	768.40	66.00	0.015	0.95
cCoastrip	Costs of one CoaguChek test strip in €	2.29	3.43	713.27	778.37	65.09	0.014	0.97
cCoasyst	Costs of CoaguChek INR device in €	537.08	805.62	718.91	772.62	53.71	0.010	0.98
noIS_Apix	No. of ischemic strokes per patient at risk and year under apixaban	0.01	0.02	735.87	785.36	49.49	0.008	0.99
noMajorBL_Apix	No. of major bleedings per patient at risk and year under apixaban	0.04	0.05	730.13	776.66	46.54	0.007	0.99
noMajorBL_War_Apix	No. of cases of major bleedings per warfarin patient at risk and year in the apibaxan trial	0.06	0.07	731.53	760.00	28.46	0.003	1.00
cMarcu	Costs of phenprocoumon per year in €	53.42	80.14	732.40	759.12	26.72	0.002	1.00
noIS_War_Apix	No. of ischemic strokes per warfarin patientsat risk and year in the apixaban trial	0.01	0.01	736.63	754.90	18.26	0.001	1.00
noHS_War_Apix	No. of hemorrhagic strokes per warfarin patient at risk and year in the apibaxan trial	0.00	0.01	739.92	751.61	11.68	0.000	1.00
noHS_Apix	No. of hemorrhagic strokes per patient at risk and year under apixaban	0.00	0.00	740.28	751.25	10.96	0.000	1.00
cMajorBL	Costs of major bleeding per event in €	3051.51	4577.27	741.91	749.62	7.72	0.000	1.00
cCoalanc	Costs of one lancet required for CoaguChek testing in €	0.14	0.22	743.71	747.82	4.10	0.000	1.00
cHStroke	Costs of hemorrhagic stroke per event in €	4384.42	6576.62	744.47	747.06	2.58	0.000	1.00
cCoalab	Costs of laboratory controls by physician for self-managing patients per year in €	2.88	4.32	745.04	746.48	1.44	0.000	1.00
cIStroke	Costs of ischemic stroke per event in €	3595.02	5938.54	745.12	746.24	1.11	0.000	1.00
cPausch	Costs of care by settled physician per year in €	90.72	136.08	745.76	745.76	0.00	0.000	1.00

*One-way sensitivity analyses of all model variables arranged in order, with the variable with the biggest impact at the top and the variable with the smallest impact at the bottom

^¥^ Risk%: This is a measure of how much of the total uncertainty is represented by the respective variable. The risk % values sum to 1.0 across all the variables.

^Ƭ^ Highest cost value minus lowest cost value

**Table 5b:** Tornado diagram*: VKA under PST versus dabigatran

**Variable Name**	**Variable Description**	**Variable lowest bound**	**Variable highest bound**	**Lowest cost value**	**Highest costs value**	**Spread^Ƭ^**	**Risk%^¥^**	**Cum Risk%**
cDabi	Drug costs of dabigatran per year in €	958,21	1437,32	472,61	951,72	479,11	0,85	0,85
pDepr	Period of depreciation	3,00	10,00	622,65	779,30	156,65	0,09	0,94
cCoatrain	Costs of training when using Coaguchek in €	84,00	150,00	668,81	734,81	66,00	0,02	0,96
cCoastrip	Costs of one CoaguChek test strips per in €	2,29	3,43	679,68	744,77	65,09	0,02	0,97
cCoasyst	Costs of CoaguChek INR device in €	537,08	805,62	685,31	739,02	53,71	0,01	0,98
noMajorBL_Dabi	No. of major bleedings per patient at risk and year underdDabigatran	0,03	0,04	695,38	728,95	33,57	0,00	0,99
noMajorBL_War_Dabi	Mo. of major bleedings per warfarin patient at risk and year in the dabigatran trial	0,03	0,04	698,31	726,03	27,72	0,00	0,99
cMarcu	Costs of phenprocoumon per year in €	53,42	80,14	698,81	725,53	26,72	0,00	0,99
noIS_War_Dabi	No. of ischemic strokes per warfarin patient at risk and year in the dabigatran trial	0,01	0,01	699,81	724,53	24,72	0,00	1,00
noIS_Dabi	No. of ischemic strokes per patient at risk and year under dabigatran	0,01	0,01	700,29	724,05	23,75	0,00	1,00
noHS_War_Dabi	No. of hemorrhagic strokes per warfarin patient at risk and year in the dabigatran trial	0,00	0,01	705,24	719,09	13,85	0,00	1,00
noHS_Dabi	No. of hemorrhagic strokes per patient at risk and year under dabigatran	0,00	0,00	707,78	716,55	8,77	0,00	1,00
cMajorBL	Costs of major bleeding per event in €	3051,51	4577,27	708,69	715,64	6,95	0,00	1,00
cHStroke	Costs of hemorrhagic stroke per event in €	4384,42	6576,62	709,97	714,36	4,39	0,00	1,00
cCoalanc	Costs of one lancet required for CoaguChek testing in €	0,14	0,22	710,12	714,22	4,10	0,00	1,00
cIStroke	Costs of ischemic stroke per event in €	3595,02	5938,54	710,68	714,20	3,53	0,00	1,00
cCoalab	Costs of laboratory controls by physician for self-managing patients per year in €	2,88	4,32	711,45	712,89	1,44	0,00	1,00
cPausch	Costs of care by settled physician per year in €	90,72	136,08	712,17	712,17	0,00	0,00	1,00

*One-way sensitivity analyses of all model variables arranged in order, with the variable with the biggest impact at the top and the variable with the smallest impact at the bottom

^¥^ Risk%: This is a measure of how much of the total uncertainty is represented by the respective variable. The risk % values sum to 1.0 across all the variables.

^Ƭ^ Highest cost value minus lowest cost value

**Table 5c:** Tornado diagram*: VKA under PST versus edoxaban

**Variable Name**	**Variable Description**	**Variable lowest bound**	**Variable highest bound**	**Lowest cost value**	**Highest costs value**	**Spread^Ƭ^**	**Risk%^¥^**	**Cum Risk%**
cEdox	Drug costs of edoxaban per year in €	870.79	1306.18	385.69	821.08	435.39	0.828	0.83
pDepr	Period of depreciation	3.00	10.00	513.87	670.51	156.65	0.107	0.94
cCoatrain	Costs of training when using Coaguchek in €	84.00	150.00	560.02	626.02	66.00	0.019	0.95
cCoastrip	Costs of one CoaguChek test strip in €	2.29	3.43	570.89	635.98	65.09	0.019	0.97
cCoasyst	Costs of CoaguChek INR device in €	537.08	805.62	576.52	630.23	53.71	0.013	0.99
noMajorBL_Edox	No. of major bleedings per patient at risk and year under edoxaban	0.02	0.03	588.88	617.87	28.99	0.004	0.99
noIS_Edox	No. of ischemic strokes per patient at risk and year under edoxaban	0.01	0.01	587.05	614.76	27.71	0.003	0.99
cMarcu	Costs of phenprocoumon per year in €	53.42	80.14	590.02	616.74	26.72	0.003	1.00
noIS_War_Edox	No. of ischemic strokes per wafarin patient at risk and year under edoxaban	0.01	0.02	591.81	614.94	23.13	0.002	1.00
noHS_War_Edox	No. of hemorrhagic strokes per warfarin patient at risk and year in the edoxaban trial	0.00	0.01	596.33	610.43	14.10	0.001	1.00
noHS_Edox	No. of hemorrhagic strokes per patient at risk and year under edoxaban	0.00	0.00	596.80	609.96	13.15	0.001	1.00
cCoalanc	Costs of one lancet required for CoaguChek testing in €	0.14	0.22	601.33	605.43	4.10	0.000	1.00
noMajorBL_War_Edox	No. of cases of major bleedings per warfarin patient at risk and year in the edoxaban trial	0.03	0.03	612.58	616.57	3.99	0.000	1.00
cIStroke	Costs of ischemic stroke per event in €	3595.02	5938.54	601.67	604.63	2.96	0.000	1.00
cHStroke	Costs of hemorrhagic stroke per event in €	4384.42	6576.62	602.09	604.67	2.59	0.000	1.00
cCoalab	Costs of laboratory controls by physician for self-managing patients per year in €	2.88	4.32	602.66	604.10	1.44	0.000	1.00
cMajorBL	Costs of major bleeding per event in €	3051.51	4577.27	603.31	603.44	0.13	0.000	1.00
cPausch	Costs of care by settled physician per year in €	90.72	136.08	603.38	603.38	0.00	0.000	1.00

*One-way sensitivity analyses of all model variables arranged in order, with the variable with the biggest impact at the top and the variable with the smallest impact at the bottom

^¥^ Risk%: This is a measure of how much of the total uncertainty is represented by the respective variable. The risk % values sum to 1.0 across all the variables.

^Ƭ^ Highest cost value minus lowest cost value

**Table 5d:** Tornado diagram*: VKA under PST versus rivaroxaban

**Variable Name**	**Variable Description**	**Variable lowest bound**	**Variable highest bound**	**Lowest cost value**	**Highest costs value**	**Spread^Ƭ^**	**Risk%^¥^**	**Cum Risk%**
cRiva	Drug costs of rivaroxaban per year in €	955.67	1433.51	523.72	1001.56	477.84	0.849	0.85
pDepr	Period of depreciation	3.00	10.00	673.13	829.77	156.65	0.091	0.94
cCoatrain	Costs of training when using Coaguchek in €	84.00	150.00	719.28	785.28	66.00	0.016	0.96
cCoastrip	Costs of one CoaguChek test strip in €	2.29	3.43	730.15	795.24	65.09	0.016	0.97
cCoasyst	Costs of CoaguChek INR device in €	537.08	805.62	735.78	789.49	53.71	0.011	0.98
noMajorBL_Riva	No. of major bleedings per patient at risk and year under rivaroxaban	0.03	0.04	744.71	780.57	35.86	0.005	0.99
noIS_Riva	No. of ischemic strokes per patient at risk and year under rivaroxaban	0.01	0.01	748.78	776.50	27.71	0.003	0.99
cMarcu	Costs of phenprocoumon per year in €	53.42	80.14	749.28	776.00	26.72	0.003	0.99
noIS_War_Riva	No. of ischemic strokes per warfarin patient at risk and year in the rivaroxaban trial	0.01	0.02	750.30	774.98	24.68	0.002	1.00
noMajorBL_War_Riva	No. of cases of major bleedings per warfarin patient at risk and year in the rivaroxaban trial	0.03	0.04	751.03	774.24	23.21	0.002	1.00
cMajorBL	Costs of major bleeding per event in €	3051.51	4577.27	752.77	772.51	19.73	0.001	1.00
noHS_Riva	No. of hemorrhagic strokes per patient at risk and year under rivaroxaban	0.00	0.00	755.51	769.76	14.25	0.001	1.00
noHS_War_Riva	No. of hemorrhagic strokes per warfarin patient at risk and year in the rivaroxaban trial	0.00	0.01	756.48	768.80	12.32	0.001	1.00
cCoalanc	Costs of one lancet required for CoaguChek testing in €	0.14	0.22	760.59	764.69	4.10	0.000	1.00
cIStroke	Costs of ischemic stroke per event in €	3595.02	5938.54	761.47	763.49	2.02	0.000	1.00
cCoalab	Costs of laboratory controls by physician for self-managing patients per year in €	2.88	4.32	761.92	763.36	1.44	0.000	1.00
cHStroke	Costs of hemorrhagic stroke per event in €	4384.42	6576.62	762.44	762.83	0.39	0.000	1.00
cPausch	Costs of care by settled physician per year in €	90.72	136.08	762.64	762.64	0.00	0.000	1.00

*One-way sensitivity analyses of all model variables arranged in order, with the variable with the biggest impact at the top and the variable with the smallest impact at the bottom

^¥^ Risk%: This is a measure of how much of the total uncertainty is represented by the respective variable. The risk % values sum to 1.0 across all the variables.

^Ƭ^ Highest cost value minus lowest cost value

**Table 6:** Results of probabilistic sensitivity analysis (Monte Carlo Simulation)

Comparators	Mean Cost per patient (€ [USD])	Standard Deviation (± SD)	Incremental Cost (€ [USD])*
Apixaban	1,608.26 [1816.53]	107.33 [121.23]	707.79 [799.45]
VKA	900.47 [1017.08]	46.08 [52.05]	0 [0]
Dabigatran	1,477.53 [1668.87]	97.42 [110.04]	719.95 [813.18]
VKA	757.58 [855.69]	41.88 [47.3]	0 [0]
Edoxaban	1,376.91 [1555.22]	89.46 [101.05]	619.2 [699.39]
VKA	757.71 [855.83]	41.06 [46.38]	0 [0]
Rivaroxaban	1,511.97 [1707.77]	99.44 [112.32]	785.24 [886.93]
VKA	726.73 [820.84]	78.52 [88.69]	0 [0]

*Incremental cost denotes the increase in total costs resulting from administering the respective NOAC versus anticoagulation with VKA (phenprocoumon) patients utilizing PST.

## Discussion

The present study is a differentiated cost-benefit analysis of the implementation of PST in VKA (phenprocoumon) patients. The cost savings in favor of VKA treatment under country-specific German conditions are predominantly attributable to the drastically higher drug costs of the 4 NOAC the annual average costs that are more than 16 (edoxaban) to 18 (apixaban) times higher compared to those of phenprocoumon with annual average costs of only €66.78 [USD 75.43].

In contrast, the risk per patient of suffering from one of the three severe adverse events (ischemic or hemorrhagic stroke or major bleeding) under VKA or NOAC is in fact only in the single-digit percent level or lower.

Increasing the TTR from the respective mean baseline level of the VKA population in the approval studies (starting from a TTR of 55%, 63% or 65%) to 78%, the realistic target of our model, nearly outweighs the initially lower number of strokes and bleedings in NOAC patients. Accordingly, the remaining differences in the associated costs compared to those incurring for severe advents in the respective NOAC groups are low. Therefore, although the costs of implementing PST, especially the costs of the INR measurement device and the patient mandatory training, in VKA patients are not negligible, NOAC drug costs remain the high cost component in our cost-benefit analysis.

Probabilistic sensitivity analysis (PSA) that considers realistic assumptions of uncertainty, demonstrates that performing PST is consistently less expensive than a NOAC. Thus, even when comprehensive discount agreements on the pharmacy retails between NOAC manufacturers and individual health insurance organizations are taken into consideration, self-managed VKA treatment can be considered the strategy of choice as far as economic aspects in favor of the German SHI are concerned.

Our study has some limitations that must be considered when interpreting our results: First, the effects of TTR increases on the number of adverse events and their associated costs are based on a mathematical approach that has been derived from a previous U.S. study without a direct relationship to the NOAC approval studies.

Second, in all NOAC approval studies warfarin was used as VKA, however, is rarely prescribed in Germany in favor of phenprocoumon. Thus, in our analysis we set warfarin and phenprocoumon, that have identical costs per tablet in Germany, to the same level of adverse effects reported in the NOAC approval studies. We should note our model may overestimate the warfarin-induced adverse effects as reported in the respective approval studies. This is because warfarin has a shorter half-life than phenprocoumon, that patients using phenprocoumon have more often INR values in therapeutic range than warfarin and thus phenprocoumon seems preferable for use in long-term therapeutic anticoagulation.^24^

Third, our calculations refer only to the treatment of strokes and major bleedings immediately after diagnosis in a German hospital. Excluded from the model are costs for aftercare services provided by special rehabilitation centers, or physiotherapeutical services offered in ambulatory outpatient settings.

Fourth, the results of the model can only be generalized if all VKA patients are mentally able to participate in PST and are willing to perform the ongoing INR checks, and that all NOAC patients regularly take their drug in the suggested doses. Indeed, a 100% implementation of PST for all patients who actually are anticoagulated with vitamin K antagonist may be a quite unrealistic option. However, in a large prospective cohort study in Switzerland those patients who decided to participate in PST had a high adherence of about 90% during a median follow-up of 4.3 years.^25^

On the other hand, there are concerns whether the results of the double-blinded approval studies of the four NOAC under consideration can also be assumed to be valid in real life: For example, in US-Veterans the adherence of AF patients to Dabigatran was reported to be only 72.2%.^26^ As the risk of a stroke increases by 13% per 10% less adherences (HR 1.13; 95% CI 1.07-1.19), an advantage with respect to adverse events simply by administering NOAC is not guaranteed.^27,28^

Thus, to validate our estimates, more cost studies, preferably with a multicenter and prospective study design, are required.

## Conclusion

The utilization of PST in anticoagulated German VKA patients with atrial fibrillation is likely to reduce overall costs. As such, routine implementation of PST may have also direct and positive impact on the control of clinical complications, especially stroke and major bleeding rates. Prospective clinical studies should be undertaken to prove our model and to further evaluate its economic advantages in the immediate future.

## Figures and Tables

**Figure:** Supplementary Content
